# Voltage-gated optics and plasmonics enabled by solid-state proton pumping

**DOI:** 10.1038/s41467-019-13131-3

**Published:** 2019-11-06

**Authors:** Mantao Huang, Aik Jun Tan, Felix Büttner, Hailong Liu, Qifeng Ruan, Wen Hu, Claudio Mazzoli, Stuart Wilkins, Chuanhua Duan, Joel K. W. Yang, Geoffrey S. D. Beach

**Affiliations:** 1 Department of Materials Science and Engineering, Massachusetts Institute of Technology, Cambridge, MA 02139 USA; 2 National Synchrotron Light Source II, Brookhaven National Laboratory, Upton, NY 11973 USA; 3 Singapore University of Technology and Design, 8 Somapah Road, Singapore, 487372 Singapore; 4 Department of Mechanical Engineering and Division of Materials Science and Engineering, Boston University, Boston, MA 02215 USA; 5 Institute of Materials Research and Engineering, A*STAR (Agency for Science, Technology and Research), 2 Fusionopolis Way, Singapore, 138634 Singapore

**Keywords:** Nanophotonics and plasmonics, Nanoscale materials, Optical materials and structures, Photonic devices, Sub-wavelength optics

## Abstract

Devices with locally-addressable and dynamically tunable optical properties underpin emerging technologies such as high-resolution reflective displays and dynamic holography. The optical properties of metals such as Y and Mg can be reversibly switched by hydrogen loading, and hydrogen-switched mirrors and plasmonic devices have been realized, but challenges remain to achieve electrical, localized and reversible control. Here we report a nanoscale solid-state proton switch that allows for electrical control of optical properties through electrochemical hydrogen gating. We demonstrate the generality and versatility of this approach by realizing tunability of a range of device characteristics including transmittance, interference color, and plasmonic resonance. We further discover and exploit a giant modulation of the effective refractive index of the gate dielectric. The simple gate structure permits device thickness down to ~20 nanometers, which can enable device scaling into the deep subwavelength regime, and has potential applications in addressable plasmonic devices and reconfigurable metamaterials.

## Abstract (web summary)

The optical properties of certain metals can be reversibly switched by hydrogen loading but challenges remain to achieve electrical and reversible control. Here, the authors report a nanoscale proton switch that allows for electrical control of optical properties through electrochemical hydrogen gating.

## Introduction

Dynamic control of optical properties by hydrogen absorption/desorption has been actively pursued since the discovery that thin metal films can be reversibly transformed into an optically-transparent state by hydrogenation^1^. Following the initial work on yttrium, hydrogen-switched optics, such as mirrors^1,2^ or other reflection-based optical devices^3,4^, were realized with a wide range of materials including Pd and Mg^5,6^. Metals such as Mg are of particular interest as they can support plasmon resonances in the visible spectrum in nanostructures with suitable geometry^6^. These resonances can enable subwavelength light manipulation, and color generation with print resolutions at the optical diffraction limit^7–9^. Hydrogen loading and unloading can serve as an effective means to change the optical response on demand to achieve active plasmonics and metasurfaces, with great potential in dynamic plasmonic color displays, dynamic holography, anti-counterfeiting technologies, and information encryption^4,10–13^. However, in most work so far, plasmonic switching is achieved through exposure to hydrogen gas in an enclosed chamber, which is impractical for many applications as it precludes addressability, i.e. local and selective switching^14^. Although hydrogen gating using ion storage layers has been achieved in solid-state tunable mirrors^15^ and similar electrochromic devices^16^, a simple, general means to locally gate hydrogenation in more complex optical and plasmonic device architectures is currently lacking.

It was recently demonstrated that a solid-state proton pump can be realized utilizing H_2_O hydrolysis in ambient atmosphere catalyzed by a GdO_x_/Au interface, where it was shown that the magnetic properties of thin buried metal layers could be gated nondestructively with a modest voltage^17^. Here, we show that a similar mechanism enables local, reversible hydrogen gating of optical properties in a wide range of metallic and plasmonic devices by covering the structure with a transparent gate oxide capped with a top electrode and applying a small voltage. We demonstrate the simplicity and utility of the mechanism by realizing nonvolatile voltage-gated color pixels based on tunable interference cells and plasmonic arrays. We further discover a large reversible change in the optical path length of the GdO_x_ upon hydrogen insertion, equivalent to a >20% change in its index of refraction, which is matched only by liquid crystal cells and complex phase-change materials^18–20^. Our approach permits switching speeds on the order of tens of ms, and device thicknesses down to several tens of nm. It promises highly localized control of a wide range of optical properties for applications such as high-resolution reflective displays, hyperspectral imaging, and dynamic holography.

## Results

### Optical property switching using a solid-state proton pump

**Fig. 1:**
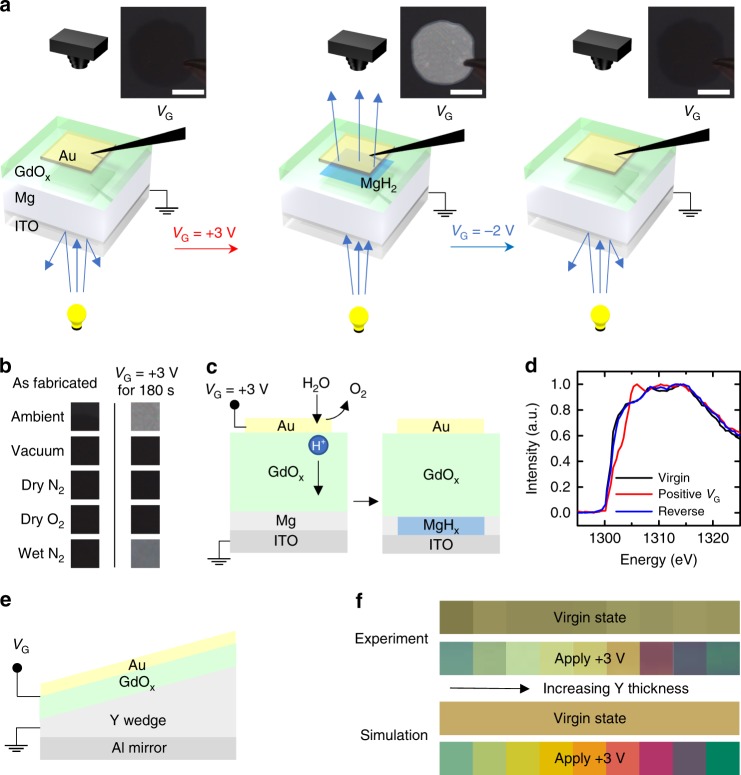
Hydrogen absorption by bottom electrode. **a** Device schematic and measurement scheme for switchable transmittance devices. The Ti and Pd layers are omitted. Transmission images are shown to the right of the camera icons. The transmission image became brighter after applying *V*_G_ = +3 V and then became darker after applying −2 V. The scale bar is 400 μm. **b** Optical images of the device as fabricated and after applying *V*_G_ = +3 V for 180 s in different gas environments (cropped to a square located at the center of the gated region). Transmission change was only observed in ambient and wet N_2_. **c** Schematic illustration of the electrochemical water splitting and Mg hydrogenation. **d** X-ray absorption spectra of Ti/Mg/Pd/GdO_x_/Au devices, in virgin state, after positive *V*_G_ application, and subsequent negative *V*_G_ application. **e** Schematic illustration of reflection device with yttrium as bottom electrode. The Ta and Pt layers are omitted. **f** Optical images and simulated colors of the devices with increasing yttrium thickness as fabricated and after applying *V*_G_ = +3 V for 180 s

We first show that a small gate voltage (*V*_G_) applied to a metal/GdO_x_/metal stack can be used to electrically switch optical transmission by injecting protons sourced from moisture in the air. Devices were formed by depositing a layer structure of Ti(3 nm)/Mg(60 nm)/Pd(5 nm)/GdO_x_(35 nm)/Au(3 nm) onto an indium tin oxide coated glass substrate (see “Methods” section), as shown schematically in Fig. 1a. Here, Mg serves as a hydrogen-switchable mirror^1,21,22^, Au serves as the top gate electrode, Pd protects the Mg from oxidation, and Ti serves as an adhesion layer. As we show below, the GdO_x_ layer catalyzes water splitting and facilitates proton conduction under a positive gate bias, allowing for voltage-gated switching of the transmissivity of the Mg mirror.

Figure 1a shows the device schematic and measurement scheme. The device was placed on a LED backlight panel and the optical images were acquired in transmission mode (Fig. 1a, insets). Due to the low transmissivity of the 60 nm thick Mg, the sample initially appears almost opaque with a barely visible darker region corresponding to the gold contact. After *V*_G_ = +3 V was applied for 180 s, a large increase in optical transmittance of the gated region was observed. When the bias is removed and the device set to open circuit, the new optical state is retained in a nonvolatile fashion. After subsequently applying *V*_G_ = −2 V for 1 h, the gated region of the transmitted image became dark again, showing that the optical switching behavior is reversible. This behavior is consistent with a reversible transition between metallic Mg and optically-transparent MgH_x_^23^, suggesting hydrogenation and dehydrogenation of Mg under gate bias. Figure 1b shows experiments performed under several different environmental conditions. Gating experiments were performed in vacuum, as well as at atmospheric pressure in ambient air, dry O_2_, dry N_2_, and wet N_2_ (see “Methods” section). The results indicate that the optical property changes only occur when there is moisture in the gas environment.

Figure 1c shows schematically the mechanism of electrochemical water splitting catalyzed at the top oxide/noble-metal interface that accounts for the reversible transition in transmittance. At positive bias, water molecules near the metal/oxide interface are split into H^+^ and O_2_. The gate bias then drives the extracted protons towards the bottom electrode, loading the Mg layer with hydrogen and switching it to an optically-transparent state. Under negative bias the reaction is reversed, leading to hydrogen removal and the return of Mg to a metallic state, consistent with the images in Fig. 1a. Figure 1d confirms this process directly, using X-ray absorption spectroscopy (XAS) to follow the chemical state change under voltage cycling (see “Methods” section). Here, the K-edge spectra of Mg from a similar device were obtained in the virgin state, after positive *V*_G_, and after subsequent negative *V*_G_ application, corresponding to the three optical states shown in Fig. 1a. The XAS spectra correspond closely to previously reported K-edge XAS spectra of metallic and hydrogenated Mg, respectively,^24^ before and after positive bias application. The spectrum recovers to the virgin state after subsequent negative bias application, showing that the hydrogen loading process is reversible. The reverse reaction only occurs in the presence of oxygen gas as shown in the previous work^17^.

We next demonstrate the generality of the device design by substituting Mg with Y in a Fabry-Pérot configuration that permits the realization of nonvolatile reflective color pixels (Fig. 1e). Here, we deposited a Ta(3 nm)/Al(100 nm)/Y(wedge 50–140 nm)/Pt(3 nm)/GdO_x_(35 nm)/Au(3 nm) film on a Si substrate (see “Methods” section). Discrete Au electrodes were fabricated across the film to allow the thickness-dependent optical properties to be probed in a single film. As fabricated, the Y layer is metallic, and devices with different yttrium thicknesses have similar reflected color as shown in Fig. 1f. After positive bias application, devices with different Y thicknesses changed to different colors as shown in Fig. 1f (bottom). Colors emerge as the metallic Y transforms into an optically-transparent dielectric that contributes to the total path length of the Fabry-Perot cavity. The total thickness of Y and GdO_x_ determines the color of the reflected light. Transfer matrix modeling (see “Methods” section) of the system agrees well with experimental results, as shown in Fig. 1f.

### Switchable plasmonic color devices

**Fig. 2:**
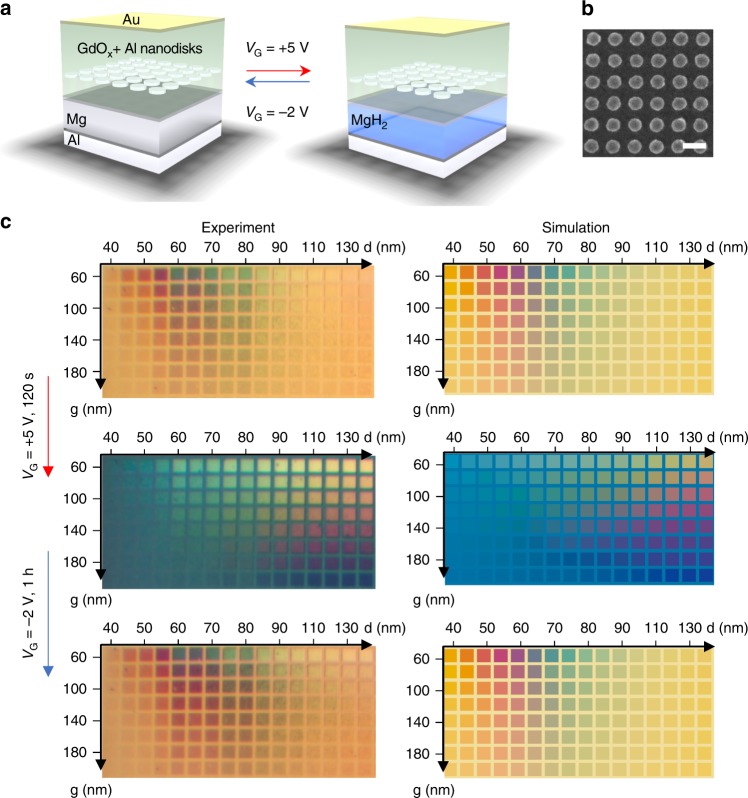
Electrochemical switchable solid-state thin film plasmonic device. **a** Schematic illustration of the switchable plasmonic device. **b** A scanning electron microscope image of the nanodisk array before 35 nm GdO_x_ and top electrode deposition. The scale bar is 200 nm. **c** Optical image (left) and simulated colors (right) of the device as fabricated, after applying +5 V for 2 mins and after applying −2 V for 1 h

To demonstrate the range of functionalities that can be achieved using this simple gating mechanism, we study switchable plasmonic color devices as shown in Fig. 2a. Starting with a uniform Al(100 nm)/Mg(40 nm)/Pd(5 nm)/GdO_x_(5 nm) film, a palette consisting of pixels of Al nanodisk arrays (Fig. 2b) was fabricated on the top (see “Methods” section). The entire set of pixels was covered by a 35 nm GdO_x_ layer and a single 3 nm Au top electrode. As shown in Fig. 2c (top), the disk diameter *d* and nearest-neighbor separation *g* were varied from array to array to yield a range of reflected colors due to the geometry-dependent plasmonic resonances. The plasmonic resonance depends on the optical properties of the surrounding layers and, therefore, can be controlled by hydrogen loading/unloading. As fabricated, Mg functions as a mirror layer below the Al disks. When a positive *V*_G_ is applied, hydrogen loading transforms the Mg into transparent MgH_x_, so that it is no longer a mirror layer. Instead, the bottom Al layer functions as the new mirror layer. Hence, the distance between the Al disks and the mirror layer increases compared to the virgin state due to the inclusion of the MgH_x_ layer. The distance increase causes a blueshift of the resonances. Optical images after applying *V*_G_ = +5 V for 120 s are shown in Fig. 2c (middle). Finite-difference time-domain (FDTD) simulations were used to model the reflected colors (see “Methods” section and Supplementary Note 1) as shown in Fig. 2c (right) which match well with the experimental results. With a later negative *V*_G_ application, the colors recovered closely to the virgin state as shown in Fig. 2c (bottom), demonstrating the reversibility of the plasmonic response control.

### Giant reversible switching of effective refractive index

**Fig. 3:**
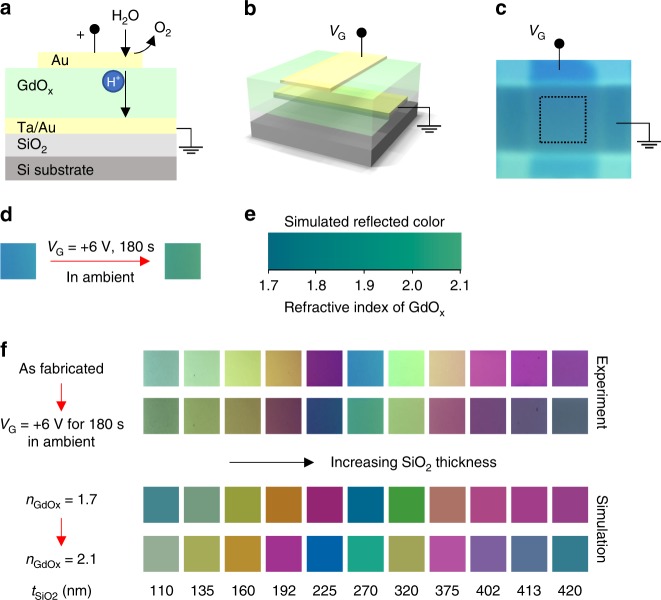
Electrochemical switchable color from solid-state thin films. **a** Schematic illustration of interference color-switching device. When a *V*_G_ > 0 is applied, water from the ambient is dissociated and the protons move towards the bottom electrode and incorporate into GdO_x_ film. **b** Schematic illustration of interference color-switching device with a crossbar structure. **c** Optical image of a typical device as fabricated. Probes are landed on the top and bottom electrode. **d** Optical images of a device with 270 nm SiO_2_ before and after voltage application in ambient. **e** Simulated reflected color of the device as a function of the refractive index of GdO_x_. **f** Optical images of devices and simulated colors with increasing SiO_2_ thickness from the left (110 nm) to the right (420 nm)

We observed that the optical properties can be switched not only through hydrogen gating of metallic and plasmonic structures, but also through large and reversible changes in the optical properties of the GdO_x_ dielectric layer itself. We demonstrate this by examining interference color switching in Ta(3 nm)/Au(3 nm)/GdO_x_(50 nm)/Au(3 nm) stacks grown on Si/SiO_2_ (${t}_{{\mathrm{SiO}}_{2}}$) and gated using a crossbar structure (Fig. 3a, b). Here, the thickness of the SiO_2_ layer (${t}_{{\mathrm{SiO}}_{2}}$) was varied from 110 nm to 420 nm, so that the reflected color due to thin-film interference varies over a wide range. The variation of ${t}_{{\mathrm{SiO}}_{2}}$ also provides multiple data points for studying the mechanism of color switching. Figure 3c shows a plan-view optical micrograph of a device with ${t}_{{\mathrm{SiO}}_{2}}$ = 270 nm in the virgin state. Figure 3d shows that the device color changes from blue to green upon positive bias application in ambient atmosphere, and Fig. 3e shows that this color change can be modeled (see “Methods” section and Supplementary Note 2) by an increase by ~0.4 of the real part of the refractive index of GdO_x_ upon hydrogen injection. Figure 3f shows that the identical index change can closely reproduce the color change observed for an array of devices with different ${t}_{{\mathrm{SiO}}_{2}}$, which act as switchable color interference devices. The observations and simulations hence suggest that a substantial change in the optical path length in the visible spectrum from voltage application is responsible for the observed color change. We attribute this effect to hydrogen accumulation in the GdO_x_ layer, which could occur through conversion to a mixed oxide-hydroxide phase changing the optical index and effective thickness, although the detailed mechanism remains to be understood. Nonetheless, the magnitude of change in effective refractive index is extremely large, comparable only to the index change achievable using liquid crystals or phase-change materials^18–20,25^. We note that the GdO_x_ refractive index change was taken into consideration in the simulations of the yttrium interference switching, and Mg plasmonic switching devices and found to play a minor role in the observed behaviors in those devices. In the plasmonic devices, the direction of the peak shift due to the change from Mg to MgH_x_ and from the increase of refractive index of GdO_x_ oppose each other, but the change from Mg to MgH_x_ dominates. The contributions from Y/Mg and GdO_x_ are compared in Supplementary Note 3.

### Switching performance

**Fig. 4:**
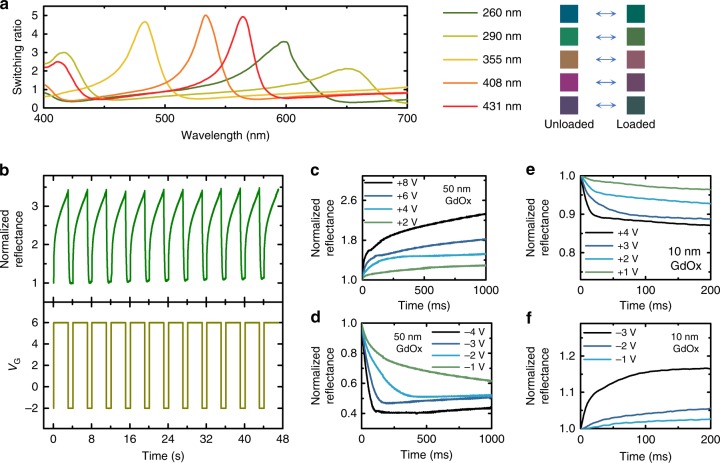
Switching ratio, reversibility and response time. **a** Reflectance switching ratio versus wavelength for five devices with different SiO_2_ thicknesses. The thickness of SiO_2_ and the color of the device in both states are shown in the legends. **b** The normalized reflectance of the 408 nm SiO_2_ sample measured by a 532 nm laser and *V*_G_ as a function of time. The reflected color can be cycled multiple times reversibly with ratio of ~3. **c**, **d** Reflectance of the optical device as a function of time measured by a 532 nm laser, showing the switching transient of a device with 50 nm GdO_x_, switching from *V*_G_ = −4 V (**c**) and from *V*_G_ = +8 V (**d**) to the voltages labeled on plots. **e**, **f** Reflectance of the optical device with 10 nm GdO_x_ and 400 nm SiO_2_ measured by a 660 nm laser as a function of time, switching from *V*_G_ = −3 V (**e**) and *V*_G_ = +4 V (**f**) to the voltages labeled on the plots

Next, we characterized the switching ratio, speed and cyclability of the voltage-gated optical devices, as these are important performance metrics for display applications. We first studied the amplitude of reflectance modulation as a function of wavelength in a series of GdO_x_ interference switching devices with different ${t}_{{\mathrm{SiO}}_{2}}$ (see “Methods” section). As shown in Fig. 4a, the reflectance modulation ratio exhibits a maximum in a wavelength range that can be tuned throughout the visible spectrum by the choice of ${t}_{{\mathrm{SiO}}_{2}}$.

We then studied the switching dynamics of the voltage-gated devices. The devices are classified into two types according to where the hydrogen is loaded. Type 1 devices include the GdO_x_ interference switching devices without a hydrogen-switched metallic layer at the bottom. In these devices, the optical modulation is due to hydrogen loading into the GdO_x_ layer. Type 2 devices have a metallic hydrogen-switched layer in the bottom, whose optical properties are modulated through voltage-gated hydrogen loading/unloading. This class of devices includes the transmission switching devices with Mg, interference switching devices with Y, and plasmonic switching devices with Mg. The switching dynamics of the two types of devices are determined by three processes: the water hydrolysis reaction at the top electrode, the transport of protons through GdO_x_, and the loading/unloading of hydrogen in the bottom metallic layer or the GdO_x_ layer. The two types of devices only differ in the third process, while they show different switching speeds. With the analysis below, we show that the gating process introduced here through the first two processes is fast, with switching time down to ~10 ms.

The switching dynamics of Type 1 devices were characterized by measuring the time-resolved reflectivity change using a 532 nm laser beam focused on the device and measuring the reflection intensity with a fast photodiode and the results are summarized in Fig. 4b–f. Here, we used the GdO_x_ interference switching device with ${t}_{{\mathrm{SiO}}_{2}}$ = 408 nm shown in Fig. 4a, whose reflectivity modulation ratio is maximum at the probe laser wavelength. Figure 4b shows the reflectivity measured while *V*_G_ was cycled between +6 V and −2 V steps at 4 s intervals. We find that the device response is reversible and asymmetric, with hydrogen unloading occurring more rapidly than loading. To quantify the switching speed, switching transients with different *V*_G_ steps were measured as the reflectance normalized by the value at the beginning of the rising/falling edge. Figure 4c shows reflectance transients for positive-going voltage steps, transitioning from *V*_G_ = −4 V to various positive values of *V*_G_. Figure 4d shows similar measurements for negative-going voltage steps transitioning from a fixed *V*_G_ = +8 V to various negative voltages. Switching transient measurements were also carried out on another device with a thinner 10 nm GdO_x_ layer (Fig. 4e, f). The switching transients were characterized by the rate of reflectivity change at the initial application of *V*_G_, and a time constant *T*_1/2_ which corresponds to the time needed to reach 50% of the maximum change (see Supplementary Note 4). As shown in Supplementary Fig. 4, the rate of reflectivity change at the initial application of *V*_G_ increases and *T*_1/2_ generally decreases as the amplitude of *V*_G_ increases, which we attribute to faster electrochemical reaction kinetics and faster proton migration through the oxide induced by larger *V*_G_. Compared to the device with a 50 nm thick GdO_x_ layer, the device with a thinner 10 nm GdO_x_ layer had shorter distance and larger electric field for proton migration. The overall smallest *T*_1/2_ of ~10 ms was achieved for the device with 10 nm GdO_x_ layer by applying *V*_G_ of +4 V/−3 V. The total device thickness responsible for the gating (excluding the Si/SiO_2_ substrate) is around 20 nm for devices with 10 nm GdO_x_. The result indicates that it is possible to achieve optical property modulation with total device thickness of ~20 nm and switching speeds on the order of 10 ms in these devices. The results suggest that the speed can potentially be further improved by replacing GdO_x_ with materials with higher proton conductivity and improved tolerance to large *V*_G_.

We then studied the switching dynamics for Type 2 devices. As discussed in Supplementary Note 5, the dynamics of Type 2 devices is more than an order of magnitude longer than Type 1 devices under the same voltage application. The result suggests that the interfacial reaction at the top electrode and the diffusion of hydrogen through the GdO_x_ layer is not limiting the speed of the switching. Instead, the hydrogenation/dehydrogenation of Mg and Y are likely to be the rate limiting step for Type 2 devices. As a result, we expect that the switching speed of Type 2 can be optimized by modifications such as adding a buffer layer between the metallic layer and the Pd capping layer^6^, using a nanostructured metallic layer to increase the surface area for hydrogenation^10^, and using hydrogen-switchable materials with faster absorption/desorption kinetics such as Mg-Ni alloy^26^ to achieve faster switching.

We also note that although the plasmonic color-switching devices shown in this work are based on hydrogen-switching of a metallic layer in the heterostructures, it is also possible to achieve plasmonic color change simply by hydrogenating the GdO_x_ layer, which is significantly faster. Supplementary Fig. 3 (left) shows that the effective refractive index change of GdO_x_ makes a significant contribution to the color change of the plasmonic devices, corresponding to color shifts by an equivalent of ~20 nm in diameter of the nanodisks. This suggests that fast plasmonic color switching can be achieved by just changing the effective refractive index of GdO_x_ via voltage gating without the rate limitation from hydrogen loading/unloading of a metallic layer.

Finally, we comment on device cyclability. As shown in Fig. 4b, the devices of Type 1 can be cycled reversibly over many cycles without significant change in device performance. In performing the measurements shown, the devices were cycled for several hundred cycles without degradation. In some devices, failure occurs after through a sudden increase in the leakage current, consistent with dielectric breakdown of the GdO_x_. We find that failures occur more frequently with larger-area devices, which is consistent with this failure mode. This observation suggests that the cyclability of the devices can potentially be optimized by improving oxide uniformity to reduce pinhole formation, or by using an electronic blocking layer in the device to minimize the electronic current. We note that voltage-gated magnetic devices using a GdO_x_ gate layer have recently been shown to be reliable for several thousand switching cycles^17^.

## Discussion

In conclusion, we demonstrated all-solid-state thin film devices with electrochemically switchable optical properties through hydrogen loading/unloading using a simple gate oxide. Reflectance, transmittance and plasmonic response gating over a wide range can be achieved by reversibly pumping hydrogen into and out of the system, based on electrocatalysis at the electrode/air interface. The active oxide can be made as thin as 10 nm so that the optical modulation can be highly localized, and high switching speeds can be achieved. The versatile system has great potential to be applied in plasmonic devices and active metamaterials to achieve control of optical property at nanoscale. Whereas we have here used GdO_x_ as a proton conducting layer, other proton conducting oxides such as Y:BaZrO_3_^27,28^ may provide even faster performance leading to ultrafast switching for displays and optical modulation applications. In addition, the ultra-thin solid-state stack allows the use of flexible substrates and realization of ultrathin pixels whose lateral dimensions can hence be scaled to deep subwavelength for high-resolution optical and holographic display applications.

## Methods

### Device fabrication

Thin film stacks were grown using magnetron sputtering at room temperature with a background pressure of ~3 × 10^−7^ Torr. Ta was deposited under 2 mTorr Ar. Au, Pt and Pd were deposited under 3.5 mTorr Ar. Al, Mg, Ti, and Y were deposited under 3 mTorr Ar. SiO_2_ layers were deposited by radio frequency (RF) sputtering under 3 mTorr Ar. GdO_x_ were deposited by RF sputtering from a Gd_2_O_3_ target under 3 mTorr Ar at an oxygen partial pressure of 0.6 mTorr. The transmission switching devices and interference devices were patterned with shadow masks. Aluminum nanodisks of the plasmonic devices were patterned with 200 nm PMMA and electron beam lithography. 25 nm of Al was deposited by electron beam evaporation, and excess material was removed by lift-off in N-methyl-2-pyrrolidone.

### Gate voltage application

Gate voltages were applied using a Keithley 2400 Sourcemeter and mechanically compliant BeCu microprobes. All gating experiments were carried out under room temperature.

### Gas ambient study

Four devices with the same device structure fabricated in the same batch were used in the study. The optical images of the devices in the virgin state were taken. Then the devices were placed in a vacuum chamber. After the vacuum level reached 10^−3^ Torr, either dry nitrogen, dry oxygen, or wet nitrogen was introduced to the chamber for each device. After the pressure level reached 20 Torr, *V*_G_ = +3 V was applied to each device for 180 s, and then the devices were left open circuit. The same +3 V, 180 s was also applied to one device at vacuum without introducing gas into the chamber. Optical transmission images of these devices were taken again after the voltage applications.

### X-ray absorption spectroscopy (XAS)

XAS data were acquired at the Coherent Soft X-ray Scattering (CSX) beamline at the National Synchrotron Light Source II, Brookhaven National Laboratory using fluorescent yield. The incident soft x-ray beam has a footprint of ~200 µm and the sample was tilted 15° relative to the incident beam. The sample used for the measurement had a crossbar geometry with about 1 mm × 1 mm gated regions and a layer structure of Ti(3 nm)/Mg(40 nm)/Pd(5 nm)/GdO_x_ (40 nm) with a 3 nm Au top gate. The main chamber base pressure was ~2 × 10^−9^ Torr, and the sample was kept at 150 K throughout the measurement. The gating was carried out ex-situ before loading into the measurement chamber. Three devices with the same structure grown side by side were used. To get the spectrum of the virgin state, no voltage application was carried out on the first device. To study the effect of positive *V*_G_, *V*_G_ = +3 V was applied for 5 min on the second device. To study the reversibility of the chemical change, *V*_G_ = −2 V was applied for 1 h after *V*_G_ = +3 V was applied for 5 min on the third device. A large color change of the devices was observed when *V*_G_ = +3 V was applied on both the second and the third device, indicating hydrogen was loaded into the third sample before the negative voltage application (hydrogen unloading).

### Optical characterization

Reflection spectra were measured using a CRAIC microspectrometer with a 20x objective and 0.5 N.A. for wavelengths between 400 and 800 nm. The measurements were calibrated with a standard Si reference.

### Switching ratio characterization

*V*_G_ = +6 V was first applied for 30 s to each device to load hydrogen, and then the device was left open circuit for 30 s while the reflection spectrum was collected. After the 30 s of open circuit, *V*_G_ = −2 V was applied for 10 s to unload hydrogen from the device. Then the device was left open circuit again and the reflection spectrum after unloading was collected. The switching ratio was calculated as the reflectance of each device after hydrogen loading over the reflectance after unloading.

### Reflected color simulation

We used transfer matrix method^29^ to simulate the reflection spectra of the interference devices. The interference color change by yttrium hydrogenation was simulated assuming yttrium changed to YH_2.9_, and Pd changed to PdH using the optical constants reported in ref. ^30^ and ref. ^31^, respectively, and assuming the refractive index of GdO_x_ changed from 1.7 to 2.1. The interference color by hydrogen loading into GdO_x_ was simulated using the GdO_x_ refractive index shown in Supplementary Fig. 1b which is adapted from ref. ^32^. The reflected spectra of plasmonic devices were simulated by FDTD method (see Supplementary Note 1 for detailed geometry). The refractive index of MgH_2_ was taken from ref. ^33^. Refractive indices of Ta, Ti, Au, Mg, and SiO_2_ from Palik^34^ were used in the simulations. The colors of the devices were rendered from reflection spectra using D65 illuminant.

## Supplementary information

Supplementary Information

## Data Availability

The data that support the findings of this study are available from the corresponding author upon reasonable request.
