# Supplementary material for: Voltage-gated optics and plasmonics enabled by solid-state proton pumping
Source: Nat Commun. 2019 Nov 6;10:5030. doi: 10.1038/s41467-019-13131-3 (PMC6834670; doi:10.1038/s41467-019-13131-3)
Supplement: Supplementary file 1 — Supplementary Information [file 41467_2019_13131_MOESM1_ESM.pdf]

Supplementary information

Voltage-gated optics and plasmonics enabled by solid-state proton pumping

Huang *et al*.

Supplementary Note 1: FDTD simulations of the switchable plasmonic colors

Finite-difference time-domain method (FDTD) simulations of switchable plasmonic color were carried out with periodic conditions along x- and y-direction and perfect matching layers along z-direction. Planer light source and frequency domain power monitor were placed above the device. The virgin device structure used in the simulation is the same as described in the main text except that the outmost 1nm in radius and the bottom and top 3nm of the aluminum nanodisks were replaced by aluminum oxide to account for oxidation that happened during fabrication. The Mg layer is assumed to expand in thickness by 30% when loaded with hydrogen[1].

Supplementary Note 2: Reflection spectrum change of devices with Si/SiO2(t_{SiO2})/Ta(3nm)/Au(3nm)/GdOx(50nm)/Au(3nm) structure

The reflectance spectra of the interference color switching devices were measured before and after V_G=+6V for 30s as shown in Supplementary Figure 1a (left). The spectra were simulated by transfer matrix method and are shown in Supplementary Figure 1a (right). The refractive index of GdO_x used in simulation is shown in Supplementary Figure 1b. The virgin state GdO_x refractive index is taken from ref[2]. The small imaginary part is ignored and assumed to stay zero when loaded with hydrogen. The simulation and experimental data show good agreement in virgin state spectrum, and in that the peaks red shift with hydrogen loading. The experimental data shows lower reflectance at shorter wavelength than simulation which is likely due to scattering caused by absorption and inhomogeneity of optical property change during voltage gating.

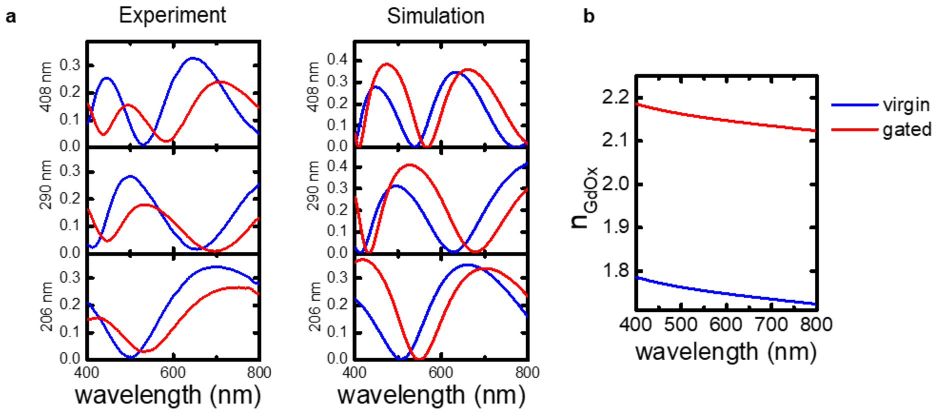

Supplementary Figure 1 a, Measured and simulated reflectance of interference color devices with three different t_{SiO2}, before and after hydrogen loading. **b**, Refractive indices of GdO_x used in the simulation (adapted from ref[2]).

Supplementary Note 3: Role of hydrogen loading to Y/Mg and GdO_x refractive index change to the optical response.

The GdO_x refractive index change was found to play a minor role in the observed behaviors in the yttrium interference switching, and Mg plasmonic switching devices. For the yttrium interference switching devices, simulations were carried out with GdO_x refractive index of 1.7 and 2.1, and the reflected colors at hydrogen loaded state are shown in Supplementary Figure 2. The minor difference in colors demonstrates that the color change with hydrogen loading is primarily due to the change of yttrium, and the refractive index change of GdO_x only contributes minor change to the colors.

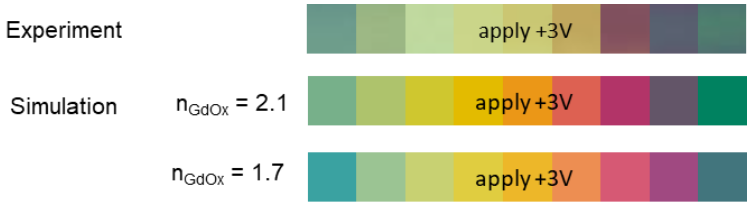

Supplementary Figure 2 Experiment micrographs and simulated colors of yttrium gating reflection devices with different GdO_x refractive index when loaded with hydrogen. The refractive index change of GdO_x only contributes minor change to the colors.

Similarly, for the Mg plasmonic switching devices, simulations were carried out with GdO_x refractive index of 1.7 and 2.1, and with Mg unloaded and loaded with hydrogen. The reflected colors are shown in Supplementary Figure 3. Comparing to Main Text Fig. 3c, the agreement to the experimental result is better with n=1.7 for the hydrogen unloaded state and n=2.1 for the loaded state. The results indicate that the plasmonic color change with hydrogen loading is primarily due to the optical change of Mg to MgH_2, and

the refractive index change of GdO_x only contributes minor change to the colors. Here we note that although the color change due to refractive index change alone is small, it is still significant to the point that the patches of colors shifted by an equivalent of ~20 nm in diameter of the nanodisks in the case of the Mg unloaded state. This suggests that plasmonic color switching devices can be made without a metallic hydrogen storage bottom layer.

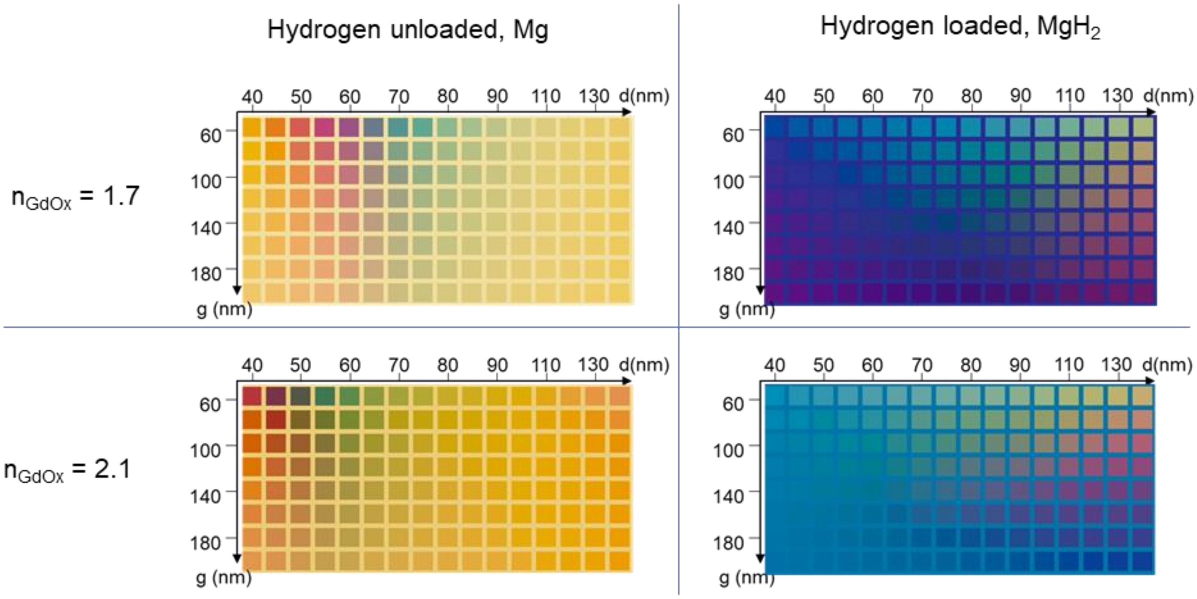

Supplementary Figure 3 Simulated colors of plasmonic switching device with different GdO_x refractive indices and different Mg hydrogen loading states. The agreement to the experimental result is better with n=1.7 for the virgin state and n=2.1 for the hydrogen loaded state.

Supplementary Note 4: Switching time dynamics of Type 1 devices

The switching time dynamics of the GdO_x interference switching devices (Type 1 devices) shown in Main Text Fig. 4 was characterized by measuring time-resolved reflectivity transients at a fixed wavelength during hydrogen loading and unloading. We find that the switching transients can be well-fitted by double-exponential functions with two separate time constants, suggesting that the rate of insertion/removal of hydrogen depends on the instantaneous concentration of loaded hydrogen: hydrogen can be rapidly pumped into an initially hydrogen-free layer, but the rate of hydrogenation decreases with time; likewise, the rate of hydrogen removal is faster immediately when a gate voltage is initially applied, and it declines with time. Although a detailed analysis of the hydrogen loading and unloading kinetics, accounting for the multiple processes involved, is beyond the present scope, we here characterized the dynamics as a function of V_G and GdO_x thickness by considering the rate of the initial transient response during hydrogen loading/unloading. In this way, we compare the rate of hydrogen insertion/removal amongst devices and bias conditions under a common hydrogen-loaded state for each measurement (either the hydrogen-depleted state for hydrogen loading measurements, or the hydrogen-saturated state for hydrogen unloading measurements.)

From the switching transients in Main Text Fig. 4, we extracted two timescales: the rate of reflectivity change at the initial application of V_G, and a time constant $T_{1/2}$ which corresponds to the time to reach 50% of the maximum observed reflectivity change. These timescales are shown are shown in Supplementary Figure 4 a,b as a function of V_G for devices with two GdO_x thicknesses. The rate of change at the initial application of V_G increases with increasing amplitude of V_G for both the 10nm and the 50nm thick GdO_x devices and for both the falling and rising edge of V_G, which we attribute to faster electrochemical reaction kinetics and faster proton migration through the oxide induced by larger V_G. With larger V_G, shorter time is needed to achieve 50% change as $T_{1/2}$. The only exception is that for the device with 50nm GdO_x, $T_{1/2}$ is

higher at positive V_G of +6V and +8V than V_G of +4V, which suggests that large positive V_G may activate the formation of a different phase that has different time dynamics. However, we caution that if the optical change does not saturate in the measurement window, then comparison of $T_{1/2}$ for different V_G may not be applicable, since the time to reach saturation at small V_G may be very much longer than the $T_{1/2}$ corresponding to the apparent saturation in the measuring time window.

The thickness of GdO_x is also an important factor in the time dynamics. A direct comparison of the rate of change at the onset of V_G is not applicable between the two types of devices because the rate of change is not only proportional to the speed of the optical property switching but also the sensitivity of the reflectivity to the optical property change determined by the optical stack and incident wavelength. The time constant on the other hand gives a better comparison for different device configurations. As shown in Supplementary Figure 4c, the time constant $T_{1/2}$ is smaller for device with thin GdO_x oxide at positive V_G edge. At the edge of negative V_G, the $T_{1/2}$ of devices with thin and thick oxide layer is comparable at -1V and -2V, and the device with 10nm GdO_x has much smaller $T_{1/2}$ than the device with 50nm GdO_x at -3V. The fastest switching ($T_{1/2}$~10ms) is achieved for the device with 10nm thick GdO_x and at V_G of +4V and -3V. Because the optical change originates from the GdO_x layer itself, a change in the GdO_x thickness also changes the amount of hydrogen that can be loaded into the device, which complicates the time dynamics comparison between devices with different GdO_x thickness.

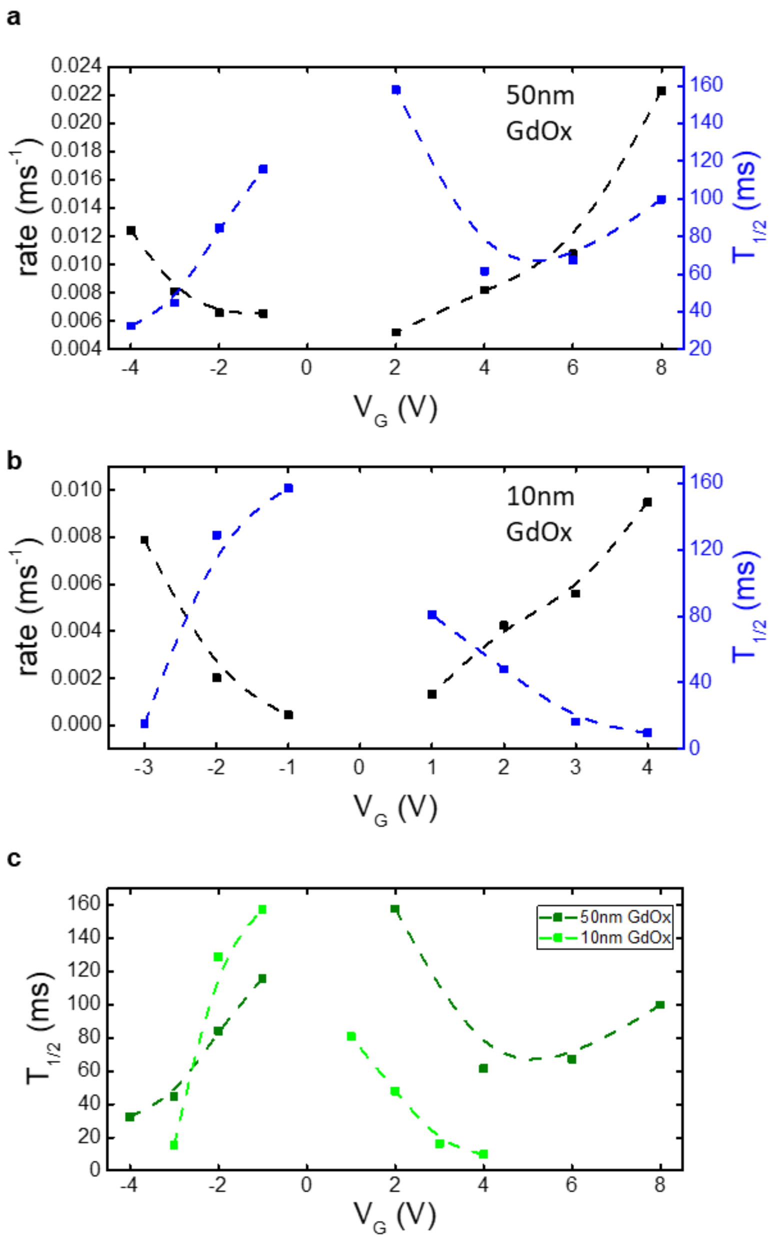

Supplementary Figure 4. Switching time dynamics of GdO_x interference device. **a** and **b**, rate of reflectivity change at the initial application of V_G and $T_{1/2}$ versus V_G for devices with GdO_x thickness of 50nm (a) and 10nm (b). **c**. $T_{1/2}$ versus V_G for devices with GdO_x thickness of 50nm and 10nm.

Supplementary Note 5: Switching speed of Mg plasmonic switching devices and comparison to GdO_x interference switching devices

We studied the switching transient of the Mg plasmonic switching devices by extracting the mean red channel intensity value from images captured from a CCD camera, using only regions without Al nanostructures. The result for V_G of +5V and -2V is shown in Supplementary Figure 5. The switching time constants $T_{1/2}$ for both the hydrogenation and dehydrogenation processes are on the order of seconds or more. In contrast, the switching time of GdO_x interference switching devices with even thicker GdO_x layer under the same V_G is around 100ms as shown in Supplementary Figure 4. Hence, the interfacial reaction at the top electrode and the diffusion of hydrogen through the GdO_x layer that are introduced by our solid-state hydrogen gating approach are not limiting the speed of the switching, but the hydrogenation/dehydrogenation of Mg and Y are likely to be the rate limiting step for the devices described in this work.

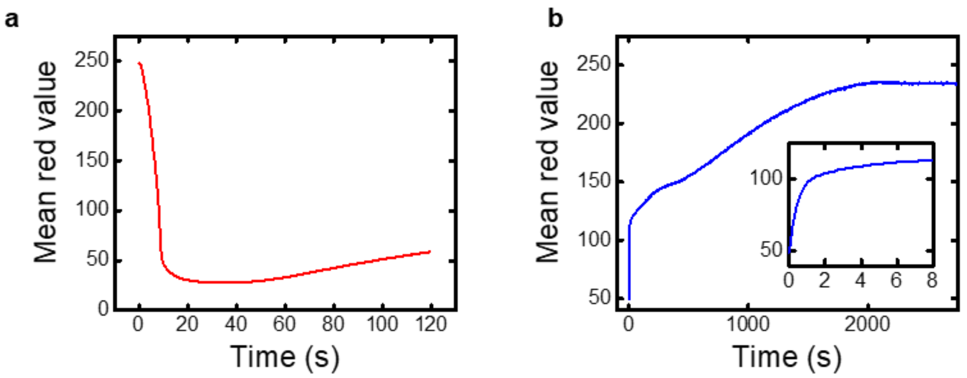

Supplementary Figure 5. Switching transient for Mg optical switching device during hydrogenation with V_G=5V (a) and dehydrogenation with V_G=-2V(b). The inset in b is the plot zoomed-in near the beginning of V_G application.

Supplementary References

1. Baldi, A., Gonzalez-Silveira, M., Palmisano, V., Dam, B. & Griessen, R. Destabilization of the mg-h system through elastic constraints. *Phys. Rev. Lett.* **102**, 1–4 (2009).

2. Dakhel, A. A. Optical constants of evaporated gadolinium oxide. *J. Opt. A Pure Appl. Opt.* **3**, 452–454 (2001).
